# Trauma-informed and relational approaches to service provision: building community-based project capacity to respond to interpersonal violence through a national initiative

**DOI:** 10.1186/s12889-020-09960-3

**Published:** 2020-11-30

**Authors:** Camilla D. Singh, Naomi C. Z. Andrews, Mary Motz, Debra J. Pepler, Margaret Leslie, Samar Zuberi

**Affiliations:** 1 Mothercraft, Early Intervention Department, 860 Richmond Street West, Toronto, Ontario M6J 1C9 Canada; 2 Department of Child and Youth Studies, Brock University, 1812 Sir Isaac Brock Way, St. Catharines, Ontario L2S 3A1 Canada; 3 Department of Psychology, York University, 4700 Keele Street, Toronto, Ontario M3J 1P3 Canada

**Keywords:** Trauma-informed principles, Interpersonal violence, Intervention, Community capacity

## Abstract

**Background:**

Community services that engage and service marginalized families can act as an important entry point for mothers and children experiencing interpersonal violence. The purpose of this study was to use an initiative that included training, implementation, and evaluation of an interpersonal violence intervention to understand changes in the capacity of service providers in community-based organizations to use trauma-informed and relational approaches to support mothers and children experiencing violence in relationships.

**Methods:**

Participants (*N* = 27) were service providers from 14 community-based organizations across Canada, who had been trained to implement an interpersonal violence intervention with mothers in their communities. Using a phenomenological approach, participants engaged in an open-ended interview in which they were encouraged to reflect on their experiences in the intervention initiative, with prompts relating to the ways in which their work and their organization may have changed as a result of their participation.

**Results:**

Findings indicated that participants were able to identify changes in four key areas related to trauma-informed practice: awareness (e.g., attitudes toward interpersonal violence), competency (e.g., application of trauma-informed knowledge), collaboration (e.g., working with other organizations to provide services to children and families), and safety (e.g., organizational policies to ensure safe, welcoming spaces). Further, participants identified these areas of change at the level of themselves as facilitators of the interpersonal violence intervention, their organizations, and their communities.

**Conclusions:**

Results indicate that, through training, implementation, and evaluation of an interpersonal violence intervention, service providers in community-based projects were able to extend trauma-informed and relational principles not only to the intervention itself, but also to their own work, as well as that of their organizations and communities. With some additional support, leveraging the existing capacities within community-based projects may be an effective means to support mothers and children experiencing interpersonal violence and other challenges.

**Supplementary Information:**

The online version contains supplementary material available at 10.1186/s12889-020-09960-3.

## Background

Interpersonal violence, or violence within relationships, is a global public health concern, estimated to affect one in three women worldwide [1]. In Canada, one quarter of all reported violent acts were related to family violence [2, 3]. This number, however, is likely low, given that it only accounts for acts of physical violence without considering other forms of interpersonal violence, such as emotional, economic, spiritual, and digital abuse [4]. Though both men and women can act as perpetrators and/or victims of interpersonal violence, women are more often victims of severe forms of violence and are more afraid of the harm that abusers cause than are men [5]. Underreporting may also contribute to underestimation of rates of interpersonal violence. Many women are reluctant to report violence for a variety of reasons, including: fear of facing the social stigma around interpersonal violence and victim blaming; fear of creating an even less safe environment for themselves and their children; and economic dependency [3, 6, 7]. The high rates of interpersonal violence point to this as a critical public health issue for women and families across Canada.

Although addressing interpersonal violence requires national public health and criminal justice efforts, communities have opportunities to promote safety for local families. Community services and supports that engage and serve vulnerable and marginalized populations can be an important entry point for women and children who may be experiencing interpersonal violence [8]. For community-based projects to successfully identify and address interpersonal violence in families within their communities, individuals must first feel comfortable and supported in a safe and caring environment. Such an environment can be achieved by intentionally applying trauma-informed and relational approaches in the context of clinical and community work with families [9, 10].

Community-based projects are often accessed by families who have been disadvantaged by unjust systems that leave them vulnerable; families often face myriad challenges including poverty, substance abuse, isolation, and parenting difficulties, among others [8, 11, 12]. Although addressing interpersonal violence may not be a central goal of these community-based projects, they are in a strong position to fill this capacity. If provided with training and ongoing support, particularly in relation to trauma-informed and relational frameworks, service providers in community-based projects are in an ideal position to create safe and nurturing environments for mothers experiencing interpersonal violence [13] (we use the terms women and mothers interchangeably because the focus of this study is women in a parenting role). The purpose of this study was to understand how participation in an initiative that included training, implementation, and evaluation of an interpersonal violence intervention enhanced the capacity of services providers in community-based parent-child organizations.

### The importance of a trauma-informed and relational approach to support families

A trauma-informed approach acknowledges the impacts of trauma on behavior and uses that knowledge to reframe and enhance services provided to avoid potential re-traumatization [4, 14]. Trauma-informed care does not require individuals to disclose a history of trauma, but rather recognizes the potential impact of trauma on current behaviour and functioning [9]. As such, utilizing a trauma-informed approach while providing services to women and children can benefit all families, whether or not they have experienced interpersonal violence [4]. This approach can manifest in community-based projects through: the creation of safe spaces and services (e.g., creating calm, stable, and predictable environments); modeling safe and healthy relationships among adults; and, for those projects serving families, promoting positive and nurturing parent-child relationships (e.g., supporting parents to help children regulate their emotions) [4, 15].

A relational approach to care involves fostering relationships in a compassionate and respectful manner to improve health outcomes [16]. This approach includes a focus on the importance of supportive, non-judgemental and compassionate relationships with clients, and developing and nurturing positive working relationships between co-workers, towards representatives from outside community agencies, and across systems [10, 15, 17]. By incorporating relational approaches in daily practice, women with a history of interpersonal violence can begin to feel safe, respected and truly supported, which may be a new experience for them in the context of their interactions with services and systems [4]. Community-based projects often engage women who have been otherwise marginalized by unjust systems. This includes systems that support gender inequality, which is recognized as a driver of family violence. Thus, service providers are in an ideal position to address interpersonal violence by learning about and incorporating these trauma-informed and relational approaches in policies and procedures, as well as through programming and daily interactions.

### The Building Connections initiative

With funding from the Public Health Agency of Canada, an initiative called *Building Connections: Supporting Community-Based Programs to Address Interpersonal Violence and Child Maltreatment* was launched by Mothercraft’s Breaking the Cycle (BTC). BTC is an early intervention and prevention program in Toronto, Canada that provides services to pregnant and parenting women using substances and their young children aged 0–6 years. The aim of Building Connections was to increase capacity among service providers in community-based projects across Canada and support them in identifying and responding to interpersonal violence, by enhancing trauma-informed and relational approaches. This initiative leveraged three existing networks of community-based projects: the Community Action Program for Children (CAPC) the Canada Prenatal Nutrition Program (CPNP), and the Aboriginal Head Start in Urban and Northern Communities (AHSUNC),^1^ all funded by the Public Health Agency of Canada [11, 12]. The overarching goals of Building Connections were: 1) to raise awareness among all CAPC/CPNP projects of effective ways to support mothers and children facing interpersonal violence and 2) to disseminate and evaluate the implementation of an interpersonal violence intervention for mothers called, *Connections: A Group Intervention for Mothers and Children Experiencing Violence in Relationships* [18] to over 30 Canadian communities where CAPC/CPNP projects are embedded.

Trauma-informed and relational principles were incorporated at each stage of this initiative. The first stage involved disseminating a resource manual (*Building Connections: Supporting Community-Based Programs to Address Interpersonal Violence and Child Maltreatment*) and a national training webinar (*Building Connections: Using Trauma-Informed and Relational Approaches to Help Women and Children Experiencing Interpersonal Violence*). These tools were designed to increase awareness and knowledge for staff from all CAPC/CPNP projects across Canada on interpersonal violence, trauma-informed/relational approaches to service provision, and application of these frameworks [4, 19]. They focused on specific ways service providers could identify and support women experiencing interpersonal violence (e.g., understanding the impact of violence on mothering and child development, prevention of trauma responses, supporting safety for women and children living with violence, trauma-informed responses to interpersonal violence).

In the second stage, staff from CAPC/CPNP projects who expressed interest in receiving more information on interpersonal violence and child maltreatment were invited to complete an application tool, *Your Starting Point Story* (YSPS) [20]. This tool was used to identify CAPC/CPNP projects with existing supports to safely deliver *Connections,* such as program policies around safety for staff and participants, the ability to provide women with services or referrals for counselling services, and working relationships with a women’s shelter and child protection services in the community. From these applications, a subset of 30 CAPC/CPNP projects were invited to send staff to attend an intensive certification training on the *Connections* intervention [19]. The training was held on-site at BTC, allowing project staff members to observe firsthand how trauma-informed, relational approaches were integrated into programming at BTC. The training itself was mostly focused on the *Connections* curriculum, highlighting both the key concepts of the intervention as well as how to (practically) deliver the intervention. The training also included explicit discussion of trauma-informed and relational approaches and how service providers might do their work in line with these approaches, as well as discussion about working through potentially difficult relationships with other community services (particularly child welfare).

While delivering the intervention and supporting the evaluation of *Connections* in their communities [10], certified facilitators continued their relationship with one another and the Building Connections team through a weekly online community of practice in which they received consultation support [19].

### Embedding trauma-informed and relational frameworks in Building Connections

The trauma-informed approaches were based on principles described by Poole [21], who indicated that best practice for clinicians and researchers should include the key principles of awareness, competency, collaboration, and safety. Specifically, best practice includes: (1) an awareness of interpersonal violence as a problem within communities and recognition of a need for intervention, (2) skills and competencies to work with families in a trauma-informed manner, (3) trauma-informed services that are well connected to supports in the community, and (4) recognition of the importance of physical and emotional safety. A relational perspective highlights that the development of people, organizations and systems within communities occurs in the context of healthy relationships [17, 22]. As such, both of these frameworks were embedded within the intervention and research processes of Building Connections, with the intention to support not only women who experience interpersonal violence, but also the certified *Connections* facilitators, who have become ambassadors of trauma-informed and relational practices within their organizations and communities [19] (see [14] for a full explanation of how these frameworks were embedded into the research processes of this initiative).

### Current study

The purpose of this study was to understand how participation in Building Connections enhanced the capacity of the certified facilitators and their organizations to engage in trauma-informed and relational work. We expected that there would be a positive impact for CAPC/CPNP projects and that embedding facilitators in this intensive experience would enable them to develop skills that were transferable to their projects and local communities, as well as influence their organizations to adopt these skill sets. Furthermore, we expected the impacts of participation in the initiative to extend beyond the CAPC/CPNP projects (herein referred to as “organization”) to other programs within the broader community that partner in service delivery (herein referred to as “community partners”). Specifically, we expected positive impacts and changes in awareness, competency, collaboration, and safety (based on key trauma-informed indicators), across facilitator, organization, and community partner levels (based on consideration of different relationships).

## Methods

### Participants

**Fig. 1:**
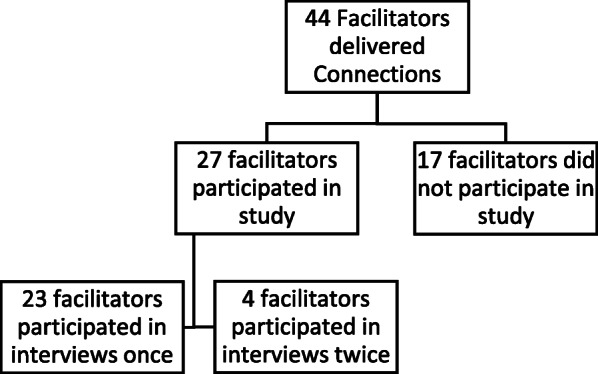
Breakdown of participants

Participants in this study were certified *Connections* facilitators from organizations involved in the Building Connections initiative who had delivered the *Connections* intervention in their home communities (participants will herein be referred to as “facilitators”). From February 2017 – July 2018, the *Connections* intervention was delivered 27 times by 44 certified facilitators in 18 communities (two facilitators always co-delivered the intervention). Upon completing the delivery of the *Connections* intervention, all facilitators were invited to participate in the current study; facilitators who ran the intervention more than once were asked to participate in interviews after each completed group intervention. Of the total 44 certified facilitators who were invited to participate, 27 facilitators from 14 organizations were interviewed (61.36% participation rate), with 4 of those 27 facilitators delivering the group and participating in two interviews (see Fig. 1). The rest of the invitations to participate in this study received no response or were declined due to scheduling difficulties. All facilitators were given the choice to participate individually or with their co-facilitator. A total of 24 interviews were conducted; 17 with individuals and 7 with two facilitators, where the two facilitators who had delivered the intervention together participated in a joint interview.

Facilitators came from diverse career backgrounds, organizations, and communities across Canada. There were no formal requirements to join the certified facilitator training; thus, the educational and professional backgrounds of facilitators varied depending on the nature of their organizations and communities. Facilitators’ positions included 10 program coordinators, 7 counselors, 6 outreach workers/home visitors, 3 intervention facilitators, and 1 executive director. Many *Connections* facilitators assumed multiple roles (e.g., working as a program coordinator as well as an outreach worker/home visitor or intervention facilitator).

Of the 14 organizations included in this study, 2 were funded through CAPC, 2 through CPNP, and 10 through both CAPC and CPNP. These organizations share a mandate to engage and support marginalized families from pregnancy to when children are 6 years old, but vary in the sociodemographic characteristics of the families they serve. The organizations operated in 8 social services agencies, 4 family resource centres, 1 early intervention program, and 1 women’s shelter. All identified poverty, food insecurity, trauma, interpersonal violence, isolation, parental substance misuse, parenting problems, and developmental delays to be challenges for the families they see. The majority also identified additional challenges in housing insecurity, parental mental health problems, child maltreatment, and Fetal Alcohol Spectrum Disorders. The organizations are embedded in communities across Canada, spanning 5 Canadian provinces (Alberta, British Columbia, Nova Scotia, Ontario, and Prince Edward Island.) Six communities are considered small population centres (population ranging from 1000 to 29,999), four are medium population centres (population ranging from 30,000-99,999), and four are large urban population centres (population of 100,000+) [23].

### Procedure

Approximately 1 month after delivering the *Connections* intervention, facilitators were invited to participate in a voluntary interview during which they were asked to reflect upon their experiences in delivering the intervention and being involved in Building Connections. Facilitators were given the options of completing the interview via telephone, online video call, or in-person (when possible). Interviews were audio-recorded and notes were taken to contextualize and facilitate transcription. Building Connections researchers alternated in moderating the interviews to reduce interviewer bias and increase validity of the data. Interviews lasted approximately 1 h. Facilitators who participated in more than one follow-up interview were provided a gift card honorarium for use within their organizations.

A phenomenological approach was used to collect and analyze data. Facilitators were asked open-ended questions, created for this study, in which they were encouraged to reflect candidly on their experiences participating in Building Connections (see Supplemental Materials). The following two questions were analyzed for the purposes of this study:

1. As a result of your participation in Building Connections, have there been any changes to the way you think about or do your work?
2. Have you suggested or supported any change in your organization? Has your involvement in building connections led to any change in your organization?

### Data analysis

#### Data preparation

Following the interviews, recordings were transcribed using an online transcription software. Transcribed interviews were edited to correct repeated words and remove filler words (e.g., use of ‘uhhh’ and ‘and’ in speech during thought), while ensuring the original meaning of the data was not lost. We used coding categories established a priori based on research related to trauma-informed approaches and relational theory. Categories included: (1) components of a trauma-informed approach (awareness, competency, collaboration, safety) and (2) level of relationships (facilitator, organization, community) [10, 21]. See Table 1 for coding guidelines and definitions.

**Table 1:** Coding guidelines and definitions

Trauma-Informed Measures		
Guidelines	Coding based on which measure change has occurred in (see definitions)	
Definitions	Awareness	Change in cognition; knowledge gained in any construct (i.e., recognizing trauma)
	Competency	Change in behaviour, application of knowledge (i.e., skill-building, modeling)
	Collaboration	Change in relationships with others, two relationships interacting (i.e., community engagement)
	Safety	Change in environment to become welcoming, consistent, reliable
Relationship Levels		
Guidelines	Coding based on who the impact, change, challenge, experience is on (see definitions)	
	Do not code if change is not occurring (i.e., conversation is about someone, but not referring to change of any sort)	
	Thoughts, ideas, plans for change should be coded under “Plans Moving Forward”	
Definitions	Facilitator	Individuals involved in the Building Connections initiative and who have delivered the curriculum
	Organization	Community-based project of which the facilitator is part
	Community	Community services and organizations outside of the project participating in Building Connections

#### Coding process

Three members of our research team (CS, NA, and MM) coded four responses together using NVivo 12 [24] to ensure coding categories were consistent across coders and representative of the data. Sentences were considered individually and categorized into trauma-informed categories (awareness, competency, collaboration, safety, null) and relational categories (facilitator, organization, community, null). Once agreement was reached, two members of the research team (CS and NA) coded two passages together and were in 100% agreement for both. They then coded six passages independently before discussing coding discrepancies. Discrepancies were resolved, and independent coding by CS and NA continued until a Coding Comparison Kappa value >.70 was reached for each of the nodes considered. After inter-rater reliability was achieved, CS coded the remainder of the interview responses.

A matrix coding query was run with NVivo software to determine the coding intersections between trauma-informed measures and relationship levels (see Table 1). Each cell of the matrix query table was reviewed by three researchers (CS, NA, MM), and unintentional categorizing due to double coding of sentences along both axes were removed. Validity in the coding categories was confirmed as all cells in the matrix coding query were well represented in the data and ensured through investigator triangulation, where three researchers independently reviewed and confirmed data was coded correctly. Each node was then summarized and categorized into themes by a researcher (MM) not primarily involved in the coding, who then established central themes based on prevalence and measured by code frequency for each theme, and confirmed with the other team members (CS and NA). Finally, this synthesis was reviewed by an additional researcher who was not part of the earlier analysis (DP), who reviewed the transcripts and the themes to further ensure that the analysis was thorough, valid and reliable.

## Results

Through interviews and focus groups, facilitators shared how their involvement in Building Connections impacted them and their work. They also reflected on the impact on their co-workers, their CAPC/CPNP projects (organizations), and community partners with whom they worked. Facilitators’ reflections were analysed to understand changes in awareness, competencies, collaborative work, and safety considerations related to mothers’ experiences of interpersonal violence. These changes are outlined below (see Table 2 for a comprehensive list of themes and frequencies; see Table 3 for frequencies by relationship levels and trauma informed measures).

**Table 2:** Observed themes and frequencies

Category		Themes	Frequency
Awareness	Facilitator	*Connections*-related constructs, perspectives, and skills (trauma-informed/relational lens)	16
		Life circumstances of participants, understanding their readiness for an interpersonal violence intervention	13
		Need for effective programming for families	9
	Organization	Identifying the need to build internal capacity and demonstrate a commitment to operating and sustaining the *Connections* intervention	27
		Recognizing unhealthy relationships/ interpersonal violence as a problem for families	9
	Community	Need to build collaborative supports and resources for families in community	9
		Recognizing the impact of interpersonal violence for families in the community	5
Competency	Facilitator	Delivering the intervention safely and effectively	15
		Use of skills and promotion of *Connections* constructs	8
	Organization	Facilitate organizational changes to be consistent with *Connections* constructs	27
		Promote, extend, and sustain *Connections* into the community	3
	Community	Develop collaborative partnerships and resources which enhance community capacity to respond to families	4
Collaboration	Facilitator	With participants – insuring the intervention is available, accessible, and safe	6
		With co-facilitators – working together effectively	3
		With community partners – building relationships, facilitating referrals, sharing knowledge and resources	5
	Organization	Organization and *Connections* facilitators working together to support the success and sustainability of the intervention	15
		Co-facilitators from different organizations working together to run the intervention	4
	Community	Sponsoring organization and community build strong and successful collaborations in the community through engagement in *Connections*	11
Safety	Facilitator	Attitudes – recognizing the need for safety in relationships with families and with the organization	4
		Behaviour – making the intervention a safe space for participants	5
	Organization	Participants – shifting organizational practices to support safe and positive relationships for families	5
		Facilitators – respectful of expertise and roles of facilitators	1
	Community	Recognizing that there is a need for interpersonal violence programming for women in the community and that filling this service gap provides important support	3
Next steps		Increase impact/support changes in own organization	7
		Increase impact/support changes in the community	6
		Pursue funding to ensure sustainability	3

**Table 3:** Frequency of coding by relationship levels and trauma informed measures

		Trauma Informed Measures			
		Awareness	Competency	Collaboration	Safety
Relationship Levels	Facilitator	38	23	14	9
	Organization	26	30	19	6
	Community	14	4	11	3

### Awareness

#### Changes for facilitators

Facilitators reported that after participating in the training and delivery of *Connections*, they had a deeper insight into trauma-informed care, relationship-based practice, and historical trauma experiences. The facilitators noted that the knowledge they gained in understanding trauma and relational practice allowed them to expand their perspectives and definitions of interpersonal violence, abuse, and trauma. One facilitator shared:

*“I always thought of IPV as with an intimate partner. With doing Connections, I’ve been able to realize that it’s not just intimate partners, it can be a sibling, it can be a parent, it can be a foster parent. It can be anyone in your life that can be an unhealthy relationship … for me, it was really restructuring and … [I acknowledge] that IPV can happen with anyone of any gender at any age at any time.”*

Facilitators also redefined what effective programming for families looked like with regard to structure, dynamics, availability, and community support (e.g., considering group readiness and goodness of fit for participant selection, individual versus group work, and closed versus open group settings). They reported being able to better identify client readiness for the *Connections* intervention and program engagement in general.

#### Changes within organizations

Within their organizations, facilitators shared observations of how staff had increased awareness of interpersonal violence as a problem for the mothers and children they served and were better able to identify unhealthy relationships. Staff also demonstrated a commitment to operating and sustaining the *Connections* intervention:

*“Connections is more on people’s radar now that it’s an official, trained program. More counsellors are often coming to check with me and say ‘Hey, I think this person really needs to do some work around this.’ It’s more a team effort, other than just me being the one that’s sort of the intake person … Everyone, even though they haven’t taken the training, … [are] flagging people who might need to do it.”*

#### Changes in the community

Facilitators noted that awareness had spread beyond their organization to community partners. According to facilitators, discussing the purpose of the *Connections* intervention with staff from community partner programs has encouraged those staff to refer clients to the *Connections* intervention. Facilitators also believed the *Connections* outreach generated increased awareness within those programs regarding potential trauma histories among the women they serve and provided a space to reflect on possible histories of abuse.

*“I talked to lots of different people from different programs and encouraged them to have these kind of conversations with the families they support. Letting them know when they’re talking to families, even if maybe that’s not the topic of conversation, they could say ‘We’re running this Connections program. It’s for women who have experienced interpersonal violence. I don’t know if that’s of interest to you … ’ Sometimes the women will say ‘Yeah, actually I’m interested in that’ and that moves that conversation that wouldn’t have come up otherwise. It opened the door and I think some workers were surprised they’ve been working with women for a little while and they weren’t aware that that was part of their life … I think people are more aware that interpersonal violence happens more often then they might think... women may not always ask for the support but if it’s offered to them, they might take it.”*

### Competency

#### Changes for facilitators

Through participation in *Connections*, facilitators reported increased competency in delivering an interpersonal violence intervention safely and effectively. They also enhanced the ways they provided services to families in their everyday work:

*“I think I notice the red flags more. I’m more confident to have those hard conversations with women … Being able to have those hard conversations and notice those red flags and be more supportive outside of facilitating because it’s just who I am. It just has made me more confident and proud that I am a trained facilitator.”*

#### Changes within organizations

Facilitators have advocated for organizational changes that are consistent with trauma-informed and relational approaches. For instance, through facilitators’ advocacy efforts, some organizations made trauma-informed changes, including managing relationships with other clients, organization staff, and with community partners, as well as improving client and staff safety through case formulation and service management planning. Facilitators reported co-workers using more trauma-informed language in their interactions with families, had an increased comfort in conversations around interpersonal violence, and learned how to respond in cases of interpersonal violence disclosure. In some cases, increased competency among staff in providing trauma-informed care resulted in changes to organizational intake and referral processes. Participation in Building Connections also increased organizational competency, as evidenced by the inclusion of material related to interpersonal violence in other programming offered within the organizations, the use of trauma-informed approaches in formal and informal interactions, and in some cases, the creation of new programming that incorporated trauma-informed and relational approaches. Certified facilitators shared their experiences delivering *Connections* with their supervisors and managers, and promoted the intervention as something that should be offered regularly, which is promising for sustainability.

*“My supervisor is very supportive of us running this program … We have to run this program almost back to back with the interest that we’re getting. She’s very supportive of that. It is part of what we do now. We run Connections. When we’re not running Connections, we have space in our calendar or our schedule to be focused on it.”*

Indeed, some facilitators have worked with their executive directors and senior leadership to reallocate existing funds and secure new funding to support ongoing delivery of *Connections,* because they found significant value in the intervention. One facilitator, an executive director in her organization, shared the following:

*“One of my goals is to re-look at our policies and procedures and even our resource manuals and to do more trauma-informed training with my staff because I have a lot of new staff now. None of them have taken any trauma-informed training. We don’t have any problem ever getting the story from our families and they’re willing to give it so how do we honour that story? How do we use it to help the family to the best of our ability? Building Connections reminded me of that other part of the story – that we have to be ready to understand.”*

Another facilitator worked tirelessly to obtain more funding to support delivering *Connections* at least three to four times a year and was successful in doing so.

*“I’m making it a priority to find a couple of extra hours of funding for it. It’s important enough and I’ve fought hard enough for it to pull money where there supposedly isn’t any, not a lot, but just a couple of hours to be able to run this more times a year. So trusting me that it is really necessary and that when there’s no money for anything and everything’s being cut, to put a couple of hours to that.”*

#### Changes in the community

Facilitators developed collaborative relationships with community partners and established resources to enhance community capacity to support vulnerable and marginalized families. Some facilitators formed co-facilitation partnerships with community partners to deliver the *Connections* intervention. Increased competencies in communities allowed facilitators to deliver the intervention in community settings such as schools, libraries, and churches through collaborative partnerships. Facilitators reported that the utilization of community spaces served to generate interest from women who typically had not accessed services within their organizations. Further, partnerships enhanced community capacity to address issues around interpersonal violence. One facilitator shared her experience of connecting with the local correctional facility:

*“I was talking to one of the supervisors at [a correctional facility] and we’ve been chatting about different types of programs, parent sessions that [my project] offers. I specifically mentioned Connections and it sounds like that’s something they’re very interested in and has offered to co-facilitate with the staff from the facility … They seem to think that they would have women that would definitely benefit from the program.”*

### Collaboration

#### Changes for facilitators

Facilitators noticed a positive change in their ability to engage clients with empathy and compassion, assess women’s readiness for the *Connections* intervention and other services, and manage referrals. Facilitators applied their learnings from Building Connections to their work in other programming, such as home visits, and used trauma-informed and relational approaches to form deeper connections with the families they served.

#### Changes within organizations

By co-facilitating the *Connections* intervention, facilitators collaborated with staff with whom they may not have previously worked. Facilitators also found that they were able to work with staff in their organization to support the success and sustainability of the intervention. Facilitators conveyed the importance of an interpersonal violence intervention to co-workers and management by sharing updates and learnings from their participation in Building Connections. These discussions led to organizational changes; for example, some organizations updated intake and referral procedures to become more trauma-informed and to improve mothers’ and children’s safety.

*“If somebody discloses domestic abuse or substance use, they then get referred immediately to me and then I kind of filter out whether they’re a good fit for the program.”*

#### Changes in the community

Facilitators established strong, successful relationships with community partner programs. They used *Connections* as a way to engage community partners and families in accessing programming within their organizations and refer clients out to community partner programs for other specialized services. These collaborations formed stronger relationships with community partners and in some cases, staff from those community partner programs were able to co-facilitate the *Connections* intervention alongside a certified *Connections* facilitator.

*“[My co-facilitator] and I doing groups together is a change. That’s something that we wouldn’t have done before so that is a change. Two programs doing a group together. They’re very separate. We’re not even in the same building area or team … And the success of it and that other teams are talking about wanting to make referrals to future groups, those kinds of things. Co-facilitating it together, two different programs, has brought about change.”*

One facilitator reflected on how enhancing collaborations with community partners had improved the quality of services clients were receiving, as service providers from various fields were able to share clinical perspectives and coordinate client care.

*“I had the realization how important it is to work with other agencies to support families and to try and see where they’re coming from as well. Why they’re making the decisions they’re making. So that’s always helpful because sometimes you don’t know all of the story.”*

### Safety

#### Changes for facilitators

Facilitators reported an increased recognition of the necessity of safety in relationships, both with families they serve, as well as with colleagues within their organizations. Facilitators discussed the importance of avoiding re-traumatizing clients; for instance, by considering the emotional readiness of women to participate in *Connections*, and by considering the attachment needs of infants and their readiness to separate from their mothers in order for mothers to attend *Connections*. Facilitators also reflected on their improved ability to recognize potential risk factors for their clients in order to make the *Connections* intervention a safe space for the women participating. Ensuring that participants felt emotionally grounded before leaving the group (particularly during weeks in which content can evoke strong emotional responses) was essential and facilitators paced the intervention based on the clients’ capacity to participate. Facilitators included opportunities for short breaks when needed, offered one-on-one debriefing after the sessions and, in some cases, extended the number of sessions in the intervention to allow more time for discussion. They also supported clients in remaining connected to their organizations and other services, both during and after the intervention, and especially when a client was in crisis. In addition to improving safety for mothers and their children, facilitators discussed the importance of ensuring their own safety (e.g., in managing their own feelings of isolation in their career, stemming from their work of treating marginalized families with complicated and traumatic histories). Facilitators appreciated having a shared community of service providers in similar situations created through Building Connections and reflected on the importance of having support while working with traumatized women and children. One facilitator noted:

*“It’s just nice to not feel as isolated doing the work we do … It’s just offered more hope I guess … there’s always potential for change or positive outcomes because sometimes when everybody’s in crisis and everybody’s having a hard time, you sometimes forget that there’s actually other families that access other programs that aren’t that way. You get tunnel vision.”*

#### Changes within organizations

In considering the safety of clients and staff, those in leadership positions within organizations have been respectful and trusting of the expertise and role of *Connections* facilitators. After seeing the positive impact of *Connections*, organizations have adopted trauma-informed and relational approaches, enhancing safe interactions and spaces for families and staff. In some organizations, referral and intake procedures were revised to incorporate a trauma-informed lens. Additionally, facilitators observed their co-workers applying concepts and using language based on trauma-informed and relational principles in their work with clients. In considering positive mother-child interactions, organizations focused on creating safe and positive transitions for mothers and children as they separated, so that mothers could attend *Connections* while their children attended childcare. Finally, facilitators described how their organizations collaborated with community partners to reduce barriers in accessing services in the community and keeping mothers and children safe.

*“It’s still protocol when someone is in need of more support than we can provide here or than we are eligible to relay, they still get referred out. But there are ways that I hope that we can do a work around, whether it’s a worker accompanying someone to a first appointment or if it’s calling the place together in that kind of peer support resourcing.”*

#### Changes in the community

Community partners recognized the need for interpersonal violence programming in their communities and the importance of filling this service gap to better support mothers and children. Facilitators found that community partners were receptive to hearing about *Connections* and agreed to work together towards a common goal of making families healthier. These agencies understood that creating a network of community supports was vital to helping marginalized women heal from their trauma and break the cycles of violence. One facilitator shared:

*“They’re going to be outreaching more. It’s going to increase outreach to other agencies and reach a population right now that is underserviced. There’s not a lot when it comes to domestic violence and kids under six and moms. I think it’s a much needed service. It reaches that population that we’re missing into the gap.”*

### Barriers and challenges

Though facilitators were overwhelmingly positive in their reports around trauma-informed and relational changes, there were also some challenges identified that may have been barriers to further changes. For instance, many facilitators have worked to make *Connections* part of the core programming in their organizations, and in some cases, as part of a continuum of programs offered. Yet, this could sometimes be challenging due to staffing changes within the organization. That is, two staff members from each community-based project was trained, but due to staff turnover, some certified facilitators ran subsequent *Connections* intervention groups with a non-trained co-facilitator. Though there were materials and resources made available for these non-certified facilitators, as well as ongoing support and training through the community of practice, staff turnover can present a challenge.

Second, a challenge both to delivery of the *Connections* intervention, but also to the potential for facilitating organizational and community change, is that service providers who work in community-based projects often fill multiple roles. There are high demands on their time and they may struggle with competing priorities. Delivering the intervention itself (including training and preparing for the group, debriefing and continued learning) adds to their workload. Advocating for trauma-informed changes to their organizations and community partner organizations also adds time and energy. Despite high levels of enthusiasm around the Building Connections initiative and the clear sense of importance of these approaches to support vulnerable women and children, timing and workload issues could sometimes present challenges.

## Discussion

Interpersonal violence is a global public health concern and can be devastating to the mental and physical health of families involved, particularly women who are at greater risk of victimization (compared to men) and their children [1]. The Building Connections initiative was designed to enhance the capacity of service providers from community-based projects to identify and respond to issues of interpersonal violence, via trauma-informed and relational approaches. Trauma-informed and relational approaches were directly taught, modeled, and embedded in all clinical, research, and evaluation activities this initiative. Results highlight the ability of service providers from community-based projects to adopt and implement trauma-informed and relational approaches in their own work, as well as extending these approaches into their organizations and the broader community, all toward the goal of supporting marginalized families in communities.

### Enhancing the capacity of community-based projects

Over and above learning how to deliver the intervention itself, facilitators were able to describe the impact that Building Connections had on: 1) their awareness and attitudes around issues of interpersonal violence; 2) their capacity and behaviours related to safely delivering trauma-informed programming in their organizations; and 3) the way that they work with others to provide service to children and families. Facilitators highlighted the importance of applying critical trauma-informed practices in their daily work (e.g., considering mothers’ trauma histories, avoiding re-traumatization) [4, 14]. They felt more confident in approaching women who may be experiencing interpersonal violence or other challenges, and more capable of building positive relationships with families in a variety of settings. Through trauma-informed and relational principles, they were able to engage with women in a manner that is safe, trustworthy, and supportive, both within the *Connections* intervention and in other programming within the organization. These increased capacities relate to the overall goal of Building Connections, which was to enhance the capacity of community-based service providers to support families who are experiencing interpersonal violence. Given that interpersonal violence is often hidden by women who are hesitant, afraid, or embarrassed to disclose violence [3, 6, 7], the capacity to identify and reach out to engage women is essential. Involvement in the Building Connections initiative has enabled service providers to reach out to women and provide appropriate support at the appropriate time. It is important to note that the certified facilitators come from various professional backgrounds, many of which are not clinical in nature. Facilitators have demonstrated that trauma-informed and relational approaches can effectively be embedded in non-clinical, community-based settings.

This research highlights that facilitators’ learning has gone beyond their own skills and capacities. Facilitators described that the training they received through Building Connections has had a positive impact on their organization and that the adoption of trauma-informed and relational practices are evident in changes in organizational attitudes and practices (e.g., changes to intake and referral processes; understanding the importance of co-facilitation). Facilitators’ involvement in Building Connections has spread within their communities, by developing new and enhanced community partnerships. Families who experience interpersonal violence face a wide range of challenges and require wrap-around support. Indeed, the experience of interpersonal violence for women can be viewed as part of a collective trauma on the basis of one’s gender, in addition to violence itself being a traumatic event. By building relationships, facilitators have brought community organizations together to support women and meet their needs in different ways. These facilitators have become ambassadors for trauma-informed and relational approaches in their organizations and communities [19].

### Limitations

There are some limitations to this study that should be noted. First, we recognize selection bias in the sample, given our screening of projects for participation. Organizations participating in Building Connections were assessed to be receptive to and capable of integrating a trauma-informed, relational intervention through the YSPS screening tool. Further, only certified facilitators who had implemented the intervention were asked to participate in the study, and only 61% of facilitators who were asked agreed to participate. Therefore, this sample of facilitators likely represents those who were most enthusiastic and perhaps capable of making important changes in their work and within their organization. Four facilitators participated in the study twice because they had delivered the intervention twice. Consequently, those who were most capable of integrating trauma-informed and relational approaches were the ones whose feedback was included and carried more weight as well as insights for the overall results.

We also recognize potential response bias, given that Building Connections researchers conducted the interviews and participants may have felt that they were expected to provide positive responses. The bias may also relate to the facilitators’ investment in the initiative with substantial and long-term involvement. We attempted to mitigate theses biases with specific instructions about providing honest answers to facilitators at multiple points during their involvement in the initiative, including at the start of the interview. We explained that facilitators’ feedback would be used to determine the effectiveness of both the *Connections* intervention and the broader Building Connections initiative, and allow Building Connections to enhance supports to marginalized mothers and children. Facilitators were reminded that no feedback would be considered as a reflection of the effectiveness (or lack thereof) of the facilitators themselves. We encouraged both positive and negative feedback.

### Implications and future directions

In the broader initiative from which this study comes, trauma-informed and relational principles were embedded as both framework and process in all aspects, including training, research, and evaluation. Identifying such positive results in terms of changes at the facilitator, organization, and community levels supports the importance of such an approach. Research has shown that it can take an average of 17 years to embed research in practice, as it requires systemic changes [25]. Results of this study show that facilitators were able to use the training and support received through the Building Connections initiative to enhance community capacity to identify and respond to interpersonal violence, within a much shorter time frame. That is, we found that service providers from across Canada reported understanding the importance of trauma-informed and relational approaches, they took up those approaches as foundational and impactful to their work, and they implemented those approaches within their own organization and throughout the community. Though promising, it remains important to consider the potential for long-term change. Specifically, are organizational policy changes or new community collaborations maintained over time? Will community collaborations continue to strengthen? Future research is needed to determine the potential long-term viability of the individual, organizational, and community-based changes found in awareness, competence, collaboration, and attention to safety.

The study also has implications for our understanding of the capacity of communities and community-based projects to support public health systems. This initiative was based within a network of federally funded prenatal and early childhood programs. These, as well as many other community-based projects, may already have structures in place to engage with marginalized families and implement trauma-informed and relational approaches. Through safe, accessible, and caring spaces, community-based projects that serve families in a variety of areas can reduce barriers to attendance, provide instrumental support (e.g., food banks, childcare), deliver programming for both parents and children, and connect families with other community supports. Community-based projects may have staff members who have already been able to establish trust with the most marginalized families. By being embedded within communities, these ‘on the ground’ service providers may be particularly receptive to and able to enhance their capacity to engage marginalized women and children with safe, relational frameworks to support the identification and prevention of interpersonal violence.

## Conclusions

This study provided evidence that through participation in a trauma-informed and relational initiative, service providers from community-based projects (CAPC and CPNP projects specifically) were able to enhance awareness, competence, collaboration, and safety in their own work, as well as in their organizations and more broadly in their communities. Results from this study support the notion that trauma-informed and relational approaches are essential to support marginalized families, and that community-based projects may be uniquely positioned to address issues of interpersonal violence. Indeed, there are networks of community-based projects across many sectors in addition to CAPC and CPNP projects (e.g., violence against women, family resource programs, shelter/housing, addictions, corrections) that have the opportunity to support the safety of mothers and children by implementing trauma-informed and relational interventions and approaches in local communities. Though there is still a vital need to address and transform systemic issues and root causes of violence against women, we found that, through trauma-informed and relational approaches, this initiative was able to leverage the existing capacities and reach within a network of community-based projects. Supporting community-based projects in this endeavor is a step towards increased identification and prevention of interpersonal violence and an important step in addressing this major public health concern.

## Supplementary Information

**Additional file 1: Table S1.** Facilitator Follow-Up Interview Questions and Prompts. The data comprise a list of questions and prompts used within interviews with facilitators. **Figure S1.** Conceptual model of the structure of the Building Connections initiative. The figure shows the people, organizations, and concepts involved in the Building Connections initiative and the structure that connects them.

## Data Availability

The datasets used and/or analyzed during the current study are available from the corresponding author on reasonable request.

## Abbreviations

BTC: Breaking the Cycle
CAPC: Community Action Program for Children
CPNP: Canada Prenatal Nutrition Program
AHSUNC: Aboriginal Head Start in Urban and Northern Communities
YSPS: Your Starting Point Story

## Footnotes

1 Staff from AHSUNC projects participated in an adapted training, implementation, and evaluation protocol, using an adapted intervention curriculum. This process will be described in a separate article; the current article focuses solely on CAPC and CPNP projects.
