# Supplementary material for: Trauma-informed and relational approaches to service provision: building community-based project capacity to respond to interpersonal violence through a national initiative
Source: BMC Public Health. 2020 Nov 30;20:1833. doi: 10.1186/s12889-020-09960-3 (PMC7706256; doi:10.1186/s12889-020-09960-3)
Supplement: Supplementary file 1 — Additional file 1: Table S1. Facilitator Follow-Up Interview Questions and Prompts. The data comprise a list of questions and prompts used within interviews with facilitators. Figure S1. Conceptual model of the structure of the Building Connections initiative. The figure shows the people, organizations, and concepts involved in the Building Connections initiative and the structure that connects them. [file 12889_2020_9960_MOESM1_ESM.docx]

Supplemental Materials

| Table 1 | |
| --- | --- |
| **Facilitator Follow-Up Interview Questions and Prompts** | |
| ***Connections* Intervention** | What was your experience like facilitating the *Connections* group? |
|  | Describe some of the successes and challenges you faced when facilitating the *Connections* intervention. |
|  | In general, was the intervention helpful to the participants? Describe why or why not, commenting on structure and content. |
|  | How do you think the women in the *Connections* group changed over the course of participating in the group with regards to their partner relationships?   1. Did you have any observations about women’s *desire/motivation* to make changes 2. Did you notice any differences in how women spoke about their relationships throughout the intervention (e.g., behavior change) 3. Did you notice any differences in how women spoke about their relationships by the end of the intervention |
|  | How do you think the women in the *Connections* group changed over the course of participating in the group with regards to their self-esteem/self-compassion/sense of the right to be safe and respected/etc.   1. Did you have any observations about women’s *desire/motivation* to make changes 2. Did you notice any differences in how women spoke about themselves throughout the intervention (e.g., behavior change) 3. Did you notice any differences in how women spoke about themselves by the end of the intervention |
|  | How do you think the women in the *Connections* group changed over the course of participating in the group with regards to their parenting?   1. Did you have any observations about women’s *desire/motivation* to make changes 2. Did you notice any differences in how women spoke about their parenting throughout the intervention (e.g., behavior change) 3. Did you notice any differences in how women spoke about their parenting by the end of the intervention |
|  | Are you currently providing services to the women who attended *Connections*? If so, have things continued to change/progress for them since the group has finished? In what ways? (relationships/self/parenting) |
|  | Did participants experience any moments of insight or discovery during the intervention? If so, please describe an example. |
|  | Did participants experience any specific or significant life events during the intervention? How did these events relate to their experience in the *Connections* group? |
|  | (If in an individual interview): How did you find facilitating the group with your co-facilitator? What were the successes and challenges you had in co-facilitating the group? |
|  | Have you worked with this person previously? Do you plan on delivering *Connections* again with the same person? Why or why not? |
|  | What else would you like us to know about your experience facilitating the *Connections* intervention? (key learnings and areas for change) |
| ***Connections* Training** | Do you have any feedback on the training (format, length, likes, dislikes)? |
|  | Did the location of the training (Breaking the Cycle) affect your overall learning experience at the training? If so, how? |
|  | How did the training affect how confident/prepared you feel to deliver the *Connections* intervention?   1. Did the training prepare you to build partnerships and/or collaborate with other community organizations? Why or why not? 2. Did the training prepare you to consider and support (i.e. provide trauma-informed services) the unique needs of families experiencing interpersonal violence? Why or why not? 3. Did the training prepare you to recruit families in your community to deliver *Connections*? Why or why not? |
| ***Connections* Community of Practice** | In general, was the *Connections* Community of Practice (CCP) helpful for you? Describe why or why not. |
|  | What else would you like us to know about your experience with the CCP? |
| **Facilitator and Organizational Change** | As a result of your participation in *Building Connections*, have there been any changes the way you think about or do your work? |
|  | Have you suggested or supported any change in your organization? Has your involvement in *Building Connections* led to any change in your organization? |


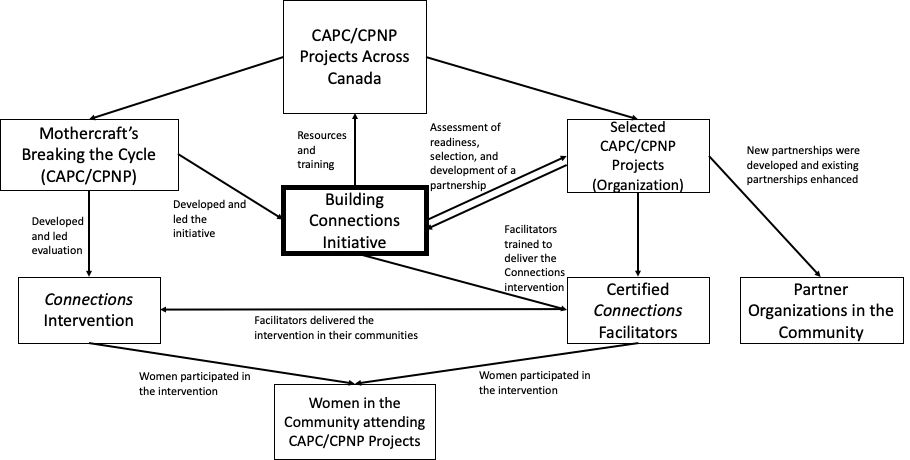


Figure 1. Conceptual model of the structure of the Building Connections initiative.
